# 基于液相色谱-串联质谱法分析血清和肽素的刺激因素

**DOI:** 10.3724/SP.J.1123.2025.05010

**Published:** 2026-03-08

**Authors:** Danni MU, Zhihong QI, Yichen MA, Yumeng GAO, Yuemeng LI, Qi ZHANG, Songlin YU, Ling QIU, Xinqi CHENG

**Affiliations:** 中国医学科学院北京协和医学院，北京协和医院检验科，北京 100730; Department of Laboratory Medicine，Peking Union Medical College Hospital，Peking Union Medical College & Chinese Academy of Medical Science，Beijing 100730，China

**Keywords:** 液相色谱-串联质谱, 和肽素, 精氨酸加压素, 精氨酸加压素缺乏症, 生长激素功能试验, liquid chromatography-tandem mass spectrometry, copeptin， arginine vasopressin, arginine vasopressin deficiency, growth hormone function test

## Abstract

和肽素作为精氨酸加压素（arginine vasopressin， AVP）的稳定替代标志物，在多尿多饮综合征的鉴别诊断中具有重要作用。当前指南建议在高渗盐水、精氨酸输注等功能刺激试验过程中动态检测和肽素水平以协助区分AVP缺乏症（AVP-D）与原发性烦渴。这些方法在诊断价值上已获得验证，但存在一定局限性。因此，寻找更安全、可操作性强且刺激效果明确的替代刺激物成为临床研究关注的方向。此外，现有的和肽素检测只有时间分辨免疫荧光法，可能受到自身抗体、溶血等干扰。本研究旨在使用实验室自建的可靠的液相色谱-串联质谱法（liquid chromatography-tandem mass spectrometry， LC-MS/MS）评估生长激素功能试验常用刺激物，包括左旋多巴、胰岛素、胰高血糖素和奥曲肽，对和肽素水平的影响。各有28、7、20和7名进行生长激素功能试验的受试者分别接受了左旋多巴、胰岛素、胰高血糖素和奥曲肽的刺激试验。在基线、30、60、90和120 min（胰高血糖素激发试验是120和180 min）时采血进行生长激素测定，使用LC-MS/MS测定各个时间点的和肽素水平。通过Wilcoxon配对符号秩检验、曼-惠特尼秩和检验和斯皮尔曼分析考察了刺激效果和相关性。研究发现，左旋多巴使和肽素水平最大增加至基线的8.47倍（*p*<0.000 1），胰岛素使其水平最大增加至基线的5.85倍（*p*=0.031 2），胰高血糖素使其水平最大增加至基线的1.43倍（*p*<0.000 1），奥曲肽使其水平最大降低至基线的43%（*p*<0.05）。和肽素水平变化与生长激素水平变化无显著相关性。在左旋多巴刺激下，AVP-D患者的和肽素最大值显著低于非AVP缺乏症患者（*p*=0.000 2），受试者工作特征曲线下面积为0.98（95%置信区间0.94~1.00，*p*=0.002 1）。本研究显示，左旋多巴和胰岛素能有效刺激和肽素分泌，而奥曲肽则抑制其分泌。LC-MS/MS检测和肽素的方法在诊断AVP-D患者中具有潜在的临床价值，可为临床提供一种新的检测手段。

## Abstract (en)

Copeptin， as a stable surrogate biomarker for arginine vasopressin （AVP）， plays an important role in the differential diagnosis of polyuria-polydipsia syndromes. Current guidelines recommend dynamic monitoring of copeptin levels during stimulation tests to assist in differentiating AVP deficiency （AVP-D） from primary polydipsia. Although these methods are well-established， they have certain limitations. Therefore， safer and more feasible stimulants with reliable effects are of clinical interest. Additionally， the currently available copeptin assay， which is based on time-resolved immunofluorescence assay， can be subject to interference from autoantibodies or hemolysis. This study aims to evaluate the effects of four common stimulants used in growth hormone function tests， namely levodopa， insulin， glucagon， and octreotide， on copeptin levels using a reliable liquid chromatography-tandem mass spectrometry （LC-MS/MS） method developed in our laboratory. A total of 62 subjects undergoing growth hormone function tests were retrospectively enrolled and stratified by stimulation type： levodopa （*n*=28）， insulin-induced hypoglycemia （*n*=7）， glucagon （*n*=20）， and octreotide （*n*=7）. Blood samples were collected at baseline， 30， 60， 90， and 120 min （for the glucagon stimulation test， samples were collected at 120 and 180 min） for growth hormone determination. Copeptin levels at each time point were measured using LC-MS/MS. The effects of stimulation and correlations were analyzed using the Wilcoxon paired signed-rank test， Mann-Whitney test， and Spearman correlation analysis. The results demonstrated that copeptin levels increased under levodopa stimulation by a maximum of 8.47-fold of baseline （*p*<0.000 1）， under insulin stimulation by a maximum of 5.85-fold of baseline （*p*=0.031 2）， under glucagon stimulation by a maximum of 1.43-fold of baseline （*p*<0.000 1）， and decreased under octreotide to 43% of baseline （*p*<0.05）. No significant correlation was observed between copeptin level changes and those in growth hormone levels. In the levodopa-stimulation group， the maximum value of copeptin in patients with AVP deficiency was significantly lower than that in non-AVP deficiency patients （*p*=0.000 2）， and the area under the receiver operating characteristic curve was 0.98 （95% confidence interval 0.94–1.00， *p*=0.002 1）. Our results demonstrate that levodopa and insulin can effectively stimulate copeptin secretion， whereas octreotide exhibits a suppressive effect. These findings offer important physiological insights into AVP regulation and indicate that certain GH stimulation agents may have extended utility in copeptin-based diagnostic strategies. The LC-MS/MS method for detecting copeptin has potential clinical value in the diagnosis of patients with AVP deficiency and can provide a new detection method for clinical practice. However， the relatively small sample sizes restrict the statistical power， and larger prospective studies are warranted.

在低渗性多尿患者中，多尿多饮综合征（polyuria-polydipsia syndrome，PPS）的识别和鉴别诊断仍然是一个难题，尤其在于如何区分精氨酸加压素（arginine vasopressin，AVP）缺乏症（AVP deficiency，AVP-D，过去称为中枢性尿崩症）与原发性烦渴（primary polydipsia，PP）^［1］^。由于各个疾病的病因不同，治疗方法不同，因此需要准确诊断。

在低渗性多尿患者的鉴别诊断中，和肽素作为AVP前体的C端片段，因其体内半衰期更长，稳定性更好，已被广泛应用为AVP的可靠替代生物标志物^［2，3］^。PPS的鉴别诊断过去依赖于禁水试验^［4］^，但已有研究和指南建议在功能刺激试验过程中动态检测和肽素水平^［1，5，6］^，以协助区分AVP-D与PP。当前推荐使用的刺激方案包括高渗盐水输注、精氨酸静脉输注等，这些方法在诊断价值上已获得验证，但部分方案存在需要静脉给药、操作复杂、刺激强度大或可引起不良反应的局限性。因此，寻找更安全、可操作性强且刺激效果明确的替代刺激物成为临床研究关注的方向。

临床上，左旋多巴、胰岛素、胰高血糖素和奥曲肽等药物常用于评估生长激素（growth hormone，GH）轴功能，具有良好的临床耐受性和应用经验基础。左旋多巴是一种口服的GH分泌激动剂，给药方便且耐受性良好。有研究表明，口服左旋多巴能显著刺激神经垂体功能正常的儿童释放和肽素^［7］^。单独使用胰岛素诱导低血糖以及联合精氨酸也能刺激和肽素分泌^［8，9］^。而胰高血糖素诱导的高血糖刺激和肽素分泌的作用有不同的研究结果^［10，11］^。这些研究提示上述刺激物亦可能影响垂体-下丘脑轴除GH外的其他激素的分泌。此外，他们还强调了需要系统评估新型刺激方法，以提高诊断准确性和患者安全性。

值得注意的是，既往研究中和肽素检测均使用时间分辨免疫荧光法^［3］^，该方法已获得欧盟认证。试剂说明书显示，线性范围2.7~2 000 pmol/L（10.85~8 040 pg/mL），检出限0.88 pmol/L（3.54 pg/mL），批内不精密度<8%。但是基于抗原抗体结合的免疫法可能受到多种干扰，如异嗜性抗体等；此外，试剂说明书中提到，溶血、脂血和黄疸可能会对检测造成干扰。由于功能试验需要连续采血（至少5个时间点），出现溶血的可能性较大，其对免疫法检测的干扰程度未知。

本实验室自建的液相色谱-串联质谱（liquid chromatography-tandem mass spectrometry，LC-MS/MS）和肽素检测法具有高灵敏度和特异度的特点，可能克服上述免疫法的局限性^［12］^。基于此，本研究拟采用实验室自建的LC-MS/MS法检测4种GH功能试验刺激物（左旋多巴、胰岛素、胰高血糖素和奥曲肽）对和肽素分泌的影响，探索其在AVP分泌功能评估中的潜在应用价值，为临床提供更简便、安全的刺激方案以及检测方案选择。

## 1 实验部分

### 1.1 仪器、试剂与材料

SCIEX Triple Quad 6500+LC-MS/MS 系统（美国AB SCIEX公司）；MIX-25混匀仪（杭州米欧仪器有限公司）；THZ-300C恒温培养摇床（上海一恒科学仪器有限公司）；正压仪、MAX 96孔固相萃取板（美国Waters公司）；氮气吹干仪（长春赛诺迈德医学技术有限公司）；IMMULITE 2000全自动化学发光免疫分析仪（德国西门子公司）； AU5800全自动生化分析仪（美国贝克曼库尔特公司）。

甲醇、乙腈（均为HPLC级），氨水（美国Thermo Fisher公司）；甲酸（美国Honeywell公司）；纯净水（屈臣氏公司）。

和肽素标准品购于美国GlpBio公司。和肽素同位素标记物（其中苯丙氨酸通过^13^C和^15^N进行标记）由上海科肽生物科技有限公司合成。和肽素抗体购于北京开景基因技术有限公司。胰蛋白酶购于美国Promega公司。

### 1.2 受试者纳入与样本收集

本研究回顾性纳入2024年8月至2025年3月在北京协和医院进行GH功能试验的患者。排除标准包括：①尿崩症，电解质紊乱以及各种急性疾病；②样本量不足或某个时间点的样本缺失；③受试者临床数据不全。该研究已通过北京协和医院伦理委员会的批准（编号：K3634），并豁免了知情同意书的签署。临床剩余血清被收集、分装至冻存管，于-80 ℃保存直至检测。

### 1.3 生长激素功能试验

左旋多巴GH兴奋试验：受试者于禁食和禁水8 h后的次日早晨开始，根据体重（<15 kg：0.125 g；15~30 kg：0.25 g；>30 kg：0.5 g），在0 min时口服左旋多巴，在0、30、60、90和120 min时采血，分离血清用于GH检测。

胰岛素低血糖GH兴奋试验：受试者禁食和禁水8 h后的次日早晨开始，空腹采血后静脉注射普通胰岛素0.1~0.15 U/kg，在30、60、90和120 min时采血，分离血清用于GH和血糖检测。

胰高血糖素激发试验：受试者禁食和禁水8 h后的次日早晨开始，根据体重（0.03 mg/kg，最大剂量1 mg）静脉注射胰高血糖素，在0、30、60、120和180 min时采血，分离血清用于GH和血糖检测。

奥曲肽敏感试验：受试者禁食和禁水8 h后的次日早晨开始，皮下注射奥曲肽0.1 mg，在0、2、4、6和8 h时采血，分离血清用于GH检测。

### 1.4 血清和肽素测定

#### 1.4.1 样品前处理

收集的血清于常温复溶、混匀后，用实验室自建的LC-MS/MS法进行检测。简而言之，1 mL血清与20 μL内标（5 ng/mL）混合后，加入100 μL抗体包被的磁珠，室温涡旋2 h让抗体充分捕获血清中的样本和内标。使用磁力架分离磁珠，去除上清。用300 μL 磷酸盐缓冲液清洗磁珠后加入30 μL 5%甲酸水溶液洗脱磁珠上的和肽素。洗脱液用24 μL 10%氨水溶液中和后，加入50 μL胰蛋白酶，在37 ℃酶切2 h。酶切后的溶液使用200 μL 10%氨水溶液稀释，加入MAX固相萃取96孔板中，分别用200 μL 10%氨水溶液和甲醇清洗，用50 μL 5%甲酸甲醇洗脱两次，洗脱液经氮气吹干后用50 μL 10%乙腈水复溶，用于上机检测。

#### 1.4.2 仪器分析

色谱柱：Waters ACQUITY UPLC^®^ Peptide BEH C18柱（100 mm×2.1 mm，1.7 μm）。流动相：A相为0.3%氨水溶液，B相为乙腈；梯度洗脱程序：0~1.5 min，10%B；1.5~4.2 min，10%B~18%B；4.2~5.5 min，18%B；5.5~5.6 min，18%B~90%B；5.6~7.0 min，90%B；7.0~8.5 min，10%B。流速0.3 mL/min；进样体积10 μＬ；柱温40 ℃；样品室温度4 ℃。

离子源：电喷雾电离源，正电离模式；扫描模式：多反应监测模式，1 007.0 → 465.2（定量离子对）；1 007.0 → 653.4（定性离子对）；1 012.0 → 465.2（内标离子对）。碰撞能30 V，去簇电压40 V，温度550 ℃，碰撞气氩气。

### 1.5 性能验证

依据临床实验室标准化协会（Clinical and Laboratory Standards Institute， CLSI）C62-A^［13］^和C64^［14］^进行验证。

20次进样时的信噪比（signal to noise， *S/N*）≥3以及变异系数（coefficient of variation， CV）≤30%的浓度被定义为检出限（limit of detection， LOD），20次进样时的 *S/N*≥10以及 CV≤20%被定义为定量限（limit of quantification， LOQ）。

对于精密度，通过将标准品添加入血清池中制备3个水平（30、100和200 pg/mL）的混合血清样本。每个样本池每天进行5次检测，连续测定5天，按照CLSI EP-15A3标准执行并计算批内不精密度（即重复性）和实验室内不精密度。

在混合血清中添加标准品（添加水平分别为 20、100 和 200 pg/mL）后，从混合血清样本中扣除初始含量后测量的和肽素浓度与添加的校准品的理论浓度进行比较，计算得出回收率。

按照样品制备程序提取6名个体的血清后，将合成的和肽素酶切后替代肽及其内标以2种浓度水平（1.25和5 ng/mL）加入其中。将标准品的绝对响应（峰面积）和分析物/内标峰面积比值与处理后血清中的响应进行比较，以评估内标是否能够校正基质效应。

### 1.6 统计学分析

连续变量和分类变量分别用中位数（25th分位数，75th分位数）和数量（百分比）来表示。使用Wilcoxon配对符号秩检验分析刺激实验中和肽素基线水平与各个时间点水平以及最大值/最小值的差异。通过斯皮尔曼分析比较和肽素与GH水平之间的相关性。使用曼-惠特尼秩和检验分析患有和不患有AVP-D的受试者在左旋多巴刺激下和肽素最大水平的差异。使用受试者工作特征曲线（receiver operating characteristic curve， ROC）分析左旋多巴刺激下和肽素最大值对于诊断AVP-D的性能。使用到的软件有Excel、IBM SPSS Statistics 27和GraphPad Prism 10.1.2。*p*<0.05表示有显著性统计学意义。

## 2 结果与讨论

### 2.1 LC-MS/MS血清和肽素测定法的性能

该方法的线性范围为5~1 000 pg/mL（*r*>0.99），检出限为2.5 pg/mL。批内不精密度（即重复性）为5.2%~12.1%，实验室内不精密度为8.1%~13.5%。回收率为95.2%~103.1%。经过内标校正后，基质效应为82.3%~118.8%。未观察到携带污染。

在本研究前期^［12］^，我们已将所建立的LC-MS/MS检测方法与当前主流的B·R·A·H·M·S KRYPTOR免疫平台进行了系统比对，发现二者在大部分浓度区间内一致性良好，Passing-Bablok回归方程的相关系数*r*=0.926（95%置信区间（CI） 0.894~0.949），斜率为0.989（95% CI 0.89~1.09），截距为1.75（95% CI -1.51~4.67）。此外，该方法不受溶血、脂血和黄疸的干扰。尽管LC-MS/MS流程相对复杂，但在特定临床应用场景下具有重要的补充价值。

### 2.2 受试者基线水平

左旋多巴GH兴奋试验、胰岛素低血糖GH兴奋试验、胰高血糖素激发试验和奥曲肽敏感试验分别纳入28、7、20和7名受试者，其年龄中位数分别为7.0、7.0、35.5和54.0岁。各组基线GH水平中位数（25th分位数，75th分位数）分别为1.1（0.2，3.07）、0.5（0.3，0.6）、2.05（1.32，5.72）和6.8（4.6，24.4） ng/mL；GH最大值的中位数分别为10.4（6.55，14.225）、8.4（2.6，12.8）和4.45（2.20，9.85） ng/mL，奥曲肽敏感试验中GH最小值的中位数为1.9（0.4，3.3） ng/mL。左旋多巴GH兴奋试验和胰岛素低血糖GH兴奋试验的受试者就诊原因均是身材矮小，其中分别有5和3人被诊断为生长激素缺乏症。

### 2.3 和肽素在功能试验中的水平变化

在左旋多巴GH兴奋试验组中，和肽素基线水平中位数（25th分位数，75th分位数）为24.40（14.05，36.40） pg/mL，服用左旋多巴后，和肽素峰值中位数为255（60.72，807.25） pg/mL，是基线水平的8.47（3.27，28.45）倍。表1显示了左旋多巴激发试验期间各时间点的和肽素水平。10名（35.71%）患者的和肽素水平在60 min时间点达到峰值，6名（21.43%）患者在120 min时达到峰值，5名（17.86%）患者的和肽素水平在30 min时间点达到峰值，而3名（10.71%）患者的和肽素水平未出现升高。根据既往研究^［15］^报道的鉴别AVP-D患者和PP患者的和肽素诊断界值3.8 pmol/L（15.276 pg/mL），27名（96.43%）患者的和肽素水平超过该界值。

**表 1:** 左旋多巴激发试验中和肽素和生长激素的水平变化

Time/min	*C*_（Copeptin）_/（pg/mL）	Copeptin fold change	*C*_（GH）_/（ng/mL）
0	24.40 （14.05， 36.40）	\	1.10 （0.20， 3.07）
30	52.00 （27.30， 734.70）^*^	1.91 （0.89， 21.00）	7.85 （2.30， 11.17）
60	144.00 （46.85， 310.75）^*^	4.82 （2.10， 13.00）	8.15 （4.27， 13.07）
90	88.40 （30.50， 196.50）^*^	2.36 （1.31， 9.23）	4.00 （1.40， 5.50）
120	71.45 （35.67， 196.50）^*^	3.33 （1.54， 8.88）	2.00 （0.72， 3.90）
Maximum	255.00 （60.72， 807.25）^*^	8.47 （3.27， 28.45）	10.40 （6.55， 14.22）

* There is a significant difference from the 0 min level （*p*<0.001）. Values are presented as median （25th， 75th percentile）.

在胰岛素低血糖GH兴奋试验中，血糖最低值的中位数为2.1（2.0，2.5） mmol/L，和肽素基线水平的中位数为23.50（17.00，62.40） pg/mL，峰值水平的中位数为200（34.60，290） pg/mL，是基线水平的5.85（1.17，12.34）倍。表2显示了胰岛素低血糖兴奋试验期间各时间点的和肽素水平。分别有2名（28.57%）患者的和肽素水平在30和60 min时间点达到峰值，而1名（14.29%）患者的和肽素水平未出现升高。根据既往研究^［15］^报道的和肽素诊断界值15.276 pg/mL，7名（100%）患者的和肽素水平均超过该界值。

**表 2:** 胰岛素低血糖兴奋试验中和肽素、生长激素和血糖的水平变化

Time/min	*C*_（Copeptin）_/（pg/mL）	Copeptin fold change	*C*_（GH）_/（ng/mL）	*C*_（Glucose）_/（mmol/L）
0	23.50 （17.00， 62.40）	\	0.50 （0.30， 0.60）	4.80 （4.80， 5.30）
30	120.00 （27.10， 245.00）	2.12 （0.92， 12.34）	4.30 （1.70， 12.80）	2.10 （2.00， 2.50）
60	90.20 （34.60， 123.00）	2.20 （1.17， 4.30）	4.30 （2.00， 11.20）	3.90 （3.40， 4.20）
90	58.30 （29.10， 136.00）	1.51 （0.98， 6.36）	2.50 （0.60， 7.00）	4.50 （3.50， 5.00）
180	51.00 （30.10， 145.00）	3.11 （0.85， 4.13）	0.70 （0.40， 2.90）	5.10 （4.40， 5.40）
Maximum	200.30 （34.60， 290.00）^*^	5.85 （1.17， 12.34）	8.40 （2.60， 12.80）	2.10（2.00，2.50）

* There is a significant difference from the 0 min level （*p*=0.0312）.

在胰高血糖素激发试验中，血糖峰值的中位数为9.25（7.85，11.72） mmol/L。和肽素基线水平的中位数为11.90（8.80，21.40） pg/mL，峰值的中位数为23.40（11.95，52.55） pg/mL，是基线水平的1.43（1.04，1.73）倍。表3显示了各时间点的和肽素水平。8名（40%）患者的和肽素水平在30 min时间点达到峰值，各有3名（15%）患者在60和180 min时达到峰值，而4名（20%）患者的和肽素水平未出现升高。根据既往研究^［15］^报道的和肽素诊断界值15.276 pg/mL，12名（60%）患者的和肽素水平在激发试验中超过该界值。

**表 3:** 胰高血糖素试验中肽素、生长激素和血糖的水平变化

Time/min	*C*_（Copeptin）_/（pg/mL）	Copeptin fold change	*C*_（GH）_/（ng/mL）	*C*_（Glucose）_/（mmol/L）
0	11.90 （8.80， 21.40）	\	2.05 （1.32， 5.72）	5.25 （4.82， 5.77）
30	16.95 （9.05， 29.80）	1.09 （0.80， 1.47）	2.35 （0.70， 5.05）	8.75 （7.72， 10.32）
60	12.00 （8.75， 31.67）	1.01 （0.75， 1.33）	3.10 （1.15， 7.10）	8.95 （7.02， 11.55）
120	12.50 （8.24， 39.82）	0.87 （0.70， 1.37）	2.20 （0.80， 4.05）	6.50 （5.55， 9.12）
180	17.10 （6.57， 288.05）	0.85 （0.61， 1.50）	2.85 （1.92， 5.35）	4.95 （3.75， 7.42）
Maximum	23.40 （11.95， 52.55）^*^	1.43 （1.04， 1.73）	4.45 （2.20， 9.85）	9.25 （7.85，11.72）

* There is a significant difference from the 0 min level （*p*<0.001）.

在奥曲肽敏感试验中，和肽素基线水平的中位数为20.50（12.90，72.90） pg/mL，最低值的中位数为6.19（5.35，31.50） pg/mL，是基线水平的0.43（0.30，0.58）倍。表4显示了各时间点的和肽素水平。各有3名（42.86%）患者的和肽素水平在2和8 h时间点达到最低值。

**表 4:** 奥曲肽抑制试验中和肽素和生长激素的水平变化

Time/h	*C*_（Copeptin）_/（pg/mL）	Copeptin fold change	*C*_（GH）_/（ng/mL）
0	20.50 （12.90， 72.90）	\	6.80 （4.60， 24.40）
2	12.10 （7.24， 31.50）^*^	0.70 （0.43， 0.92）	1.90 （0.50， 4.10）
4	16.70 （14.50， 63.30）	0.87 （0.75， 1.09）	2.50 （0.40， 3.80）
6	18.10 （5.85， 58.80）	0.81 （0.47， 0.88）	3.30 （0.80， 3.90）
8	11.30 （6.19， 40.30）^*^	0.57 （0.30， 0.76）	3.40 （0.60， 5.00）
Minimum	6.19 （5.35， 31.50）^*^	0.43 （0.30， 0.58）	1.90 （0.40， 3.30）

* There is a significant difference from the 0 min level （*p*<0.05）.

AVP功能障碍可能导致一系列液体紊乱，包括PPS和抗利尿激素分泌异常综合征等^［16］^。根据病因，PPS可分为三类：①AVP-D（过去称为中枢性尿崩症），其特点是下丘脑和垂体后叶AVP的合成和分泌不足；②AVP抵抗（过去称为肾性尿崩症），表现为肾脏对AVP的敏感性不足；③PP，其特点是液体摄入过多^［17］^。为实现正确有效的治疗，准确鉴别这些疾病至关重要^［18］^。PPS的诊断需要结合病史、实验室信息（如血钠和渗透压等）、磁共振成像、禁水试验结果等综合判断。

近年来，和肽素在PPS诊断中的作用被广泛肯定。目前，间接禁水试验、高渗盐水输注试验、精氨酸输注试验、胰高血糖素刺激试验均被证明有刺激和肽素分泌的作用，并且在此过程中检测和肽素能鉴别AVP-D和PP患者^［10，19，20］^。Refardt等^［21］^的一项头对头实验显示，高渗盐水刺激优于精氨酸输注试验，但72%的患者偏好更简单的精氨酸刺激，因为不良反应更少且更轻微。但精氨酸并不广泛可用，限制了其临床应用；此外，有因高浓度的精氨酸外渗导致皮肤坏死的病例报道^［22］^。因此寻找更多的有效且安全的刺激物有重要临床价值。近期的研究显示，左旋多巴、胰岛素和尿素也能引起和肽素的分泌^［7-9，23］^。对于口服左旋多巴，有研究报道了儿童群体中和肽素水平最高可以升高3.94倍^［7］^；但也有研究称，左旋多巴不能刺激和肽素分泌^［24］^。在胰岛素诱导的低血糖试验中，既往研究报道了和肽素水平相较于基线增加了1.32~1.90倍^［8，25，26］^；联合精氨酸和胰岛素可以诱发更强烈的刺激反应^［9］^。而可乐定和马西瑞林对其分泌无显著影响^［25，27］^。本研究呈现出与既往文献相似的结果，即左旋多巴和胰岛素能显著刺激和肽素分泌，胰高血糖素刺激的效果较小。此外，我们首次发现奥曲肽有抑制和肽素分泌的作用。

左旋多巴作为一种多巴胺前体，能够刺激多巴胺能和肾上腺素能神经元使得GH分泌^［28］^，其对神经垂体功能的影响在既往文献中亦有报道，然而其刺激效应的作用机制仍不明确^［7］^。既往研究还报道左旋多巴可刺激促肾上腺皮质激素和皮质醇分泌，这提示多巴胺能系统对下丘脑-垂体-肾上腺（hypothalamic-pituitary-adrenal axis， HPA）轴具有正向调控作用^［29］^。因此，左旋多巴给药后和肽素的释放是否涉及HPA轴激活仍需进一步探究。胰岛素可诱导低血糖，激发强烈的生理应激反应；胰高血糖素同理，但其刺激作用较弱。相关研究表明，在应激状态下，AVP、和肽素与促肾上腺皮质激素释放激素（corticotropin releasing hormone， CRH）可能被共同释放^［30-32］^。这可能解释了本研究中胰岛素和胰高血糖素对和肽素的刺激现象，且前者诱发的低血糖有更强烈的效果。奥曲肽可抑制垂体分泌GH或促甲状腺激素（thyroid stimulating hormone， TSH），用于判断GH腺瘤或TSH腺瘤患者对生长抑素类似物治疗的反应，其对和肽素的抑制作用在本研究中被报道。我们推测，奥曲肽可能间接抑制了合成AVP前体蛋白的大细胞神经元或小细胞神经元的兴奋性，也可能直接抑制垂体后叶释放激素的能力。这一结果为今后深入探讨AVP调控通路提供了新的线索。

需要指出的是，各时间点和肽素水平的误差线较大，反映了不同受试者在刺激试验中的个体差异性。这种变异主要来自两个方面：一方面，和肽素水平受多种生理因素影响，包括血浆渗透压、循环容量、脱水状态、应激状态以及试验前饮水或禁水的情况^［5］^，可能导致基线水平及刺激反应幅度在不同个体之间存在显著差异。另一方面，本研究采用的LC-MS/MS方法具有较高的灵敏度和特异性，但由于其样本处理流程涉及免疫富集、酶解、固相萃取等多个步骤，操作中的微小差异亦可能引入变异。尤其是在低浓度区间，测量不确定性相对更高，也可能导致偏差的放大。综上所述，结果呈现的误差范围是在可接受的生理和方法学波动范围内，反映了和肽素在真实临床样本中的自然分布特征，亦支持其在功能试验中作为动态反应指标的临床应用潜力。

### 2.4 和肽素水平变化与生长激素水平变化的相关性

在4项功能试验中，和肽素峰值与生长激素峰值之间均不存在显著相关性（图1），两者的变化倍数之间也不存在显著相关（图2），两者的达峰（或达谷）时间也无显著相关性（图3）。

**图1:**
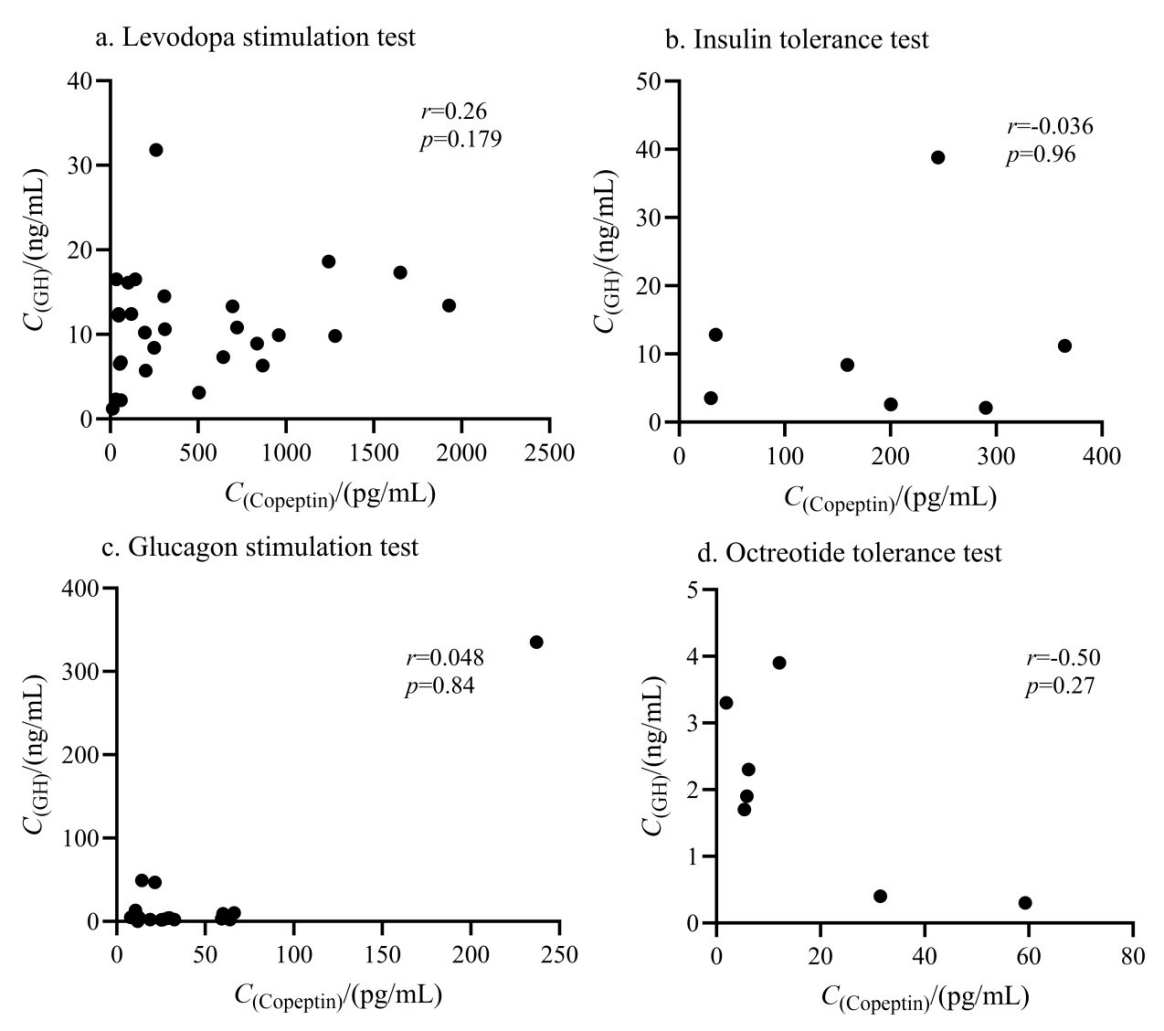
（a）左旋多巴激发试验（*n*=28）、（b）胰岛素低血糖兴奋试验（*n*=7）、（c）胰高血糖素激发试验（*n*=20）和（d）奥曲肽抑制试验（*n*=7）期间GH最值与和肽素最值之间的相关性

**图2:**
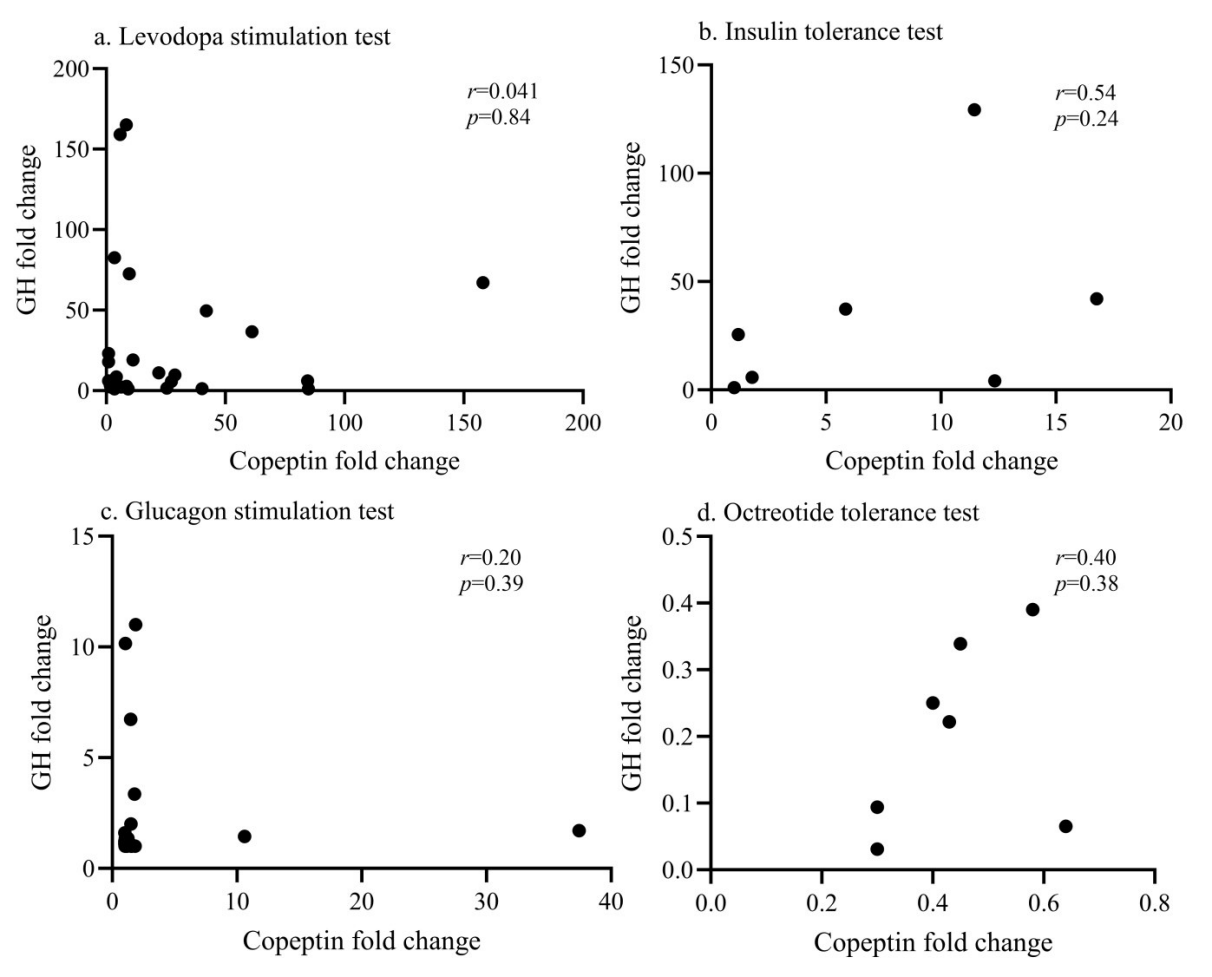
（a）左旋多巴激发试验（*n*=28）、（b）胰岛素低血糖兴奋试验（*n*=7）、（c）胰高血糖素激发试验（*n*=20）和（d）奥曲肽抑制试验（*n*=7）期间GH变化倍数与和肽素变化倍数之间的相关性

**图3:**
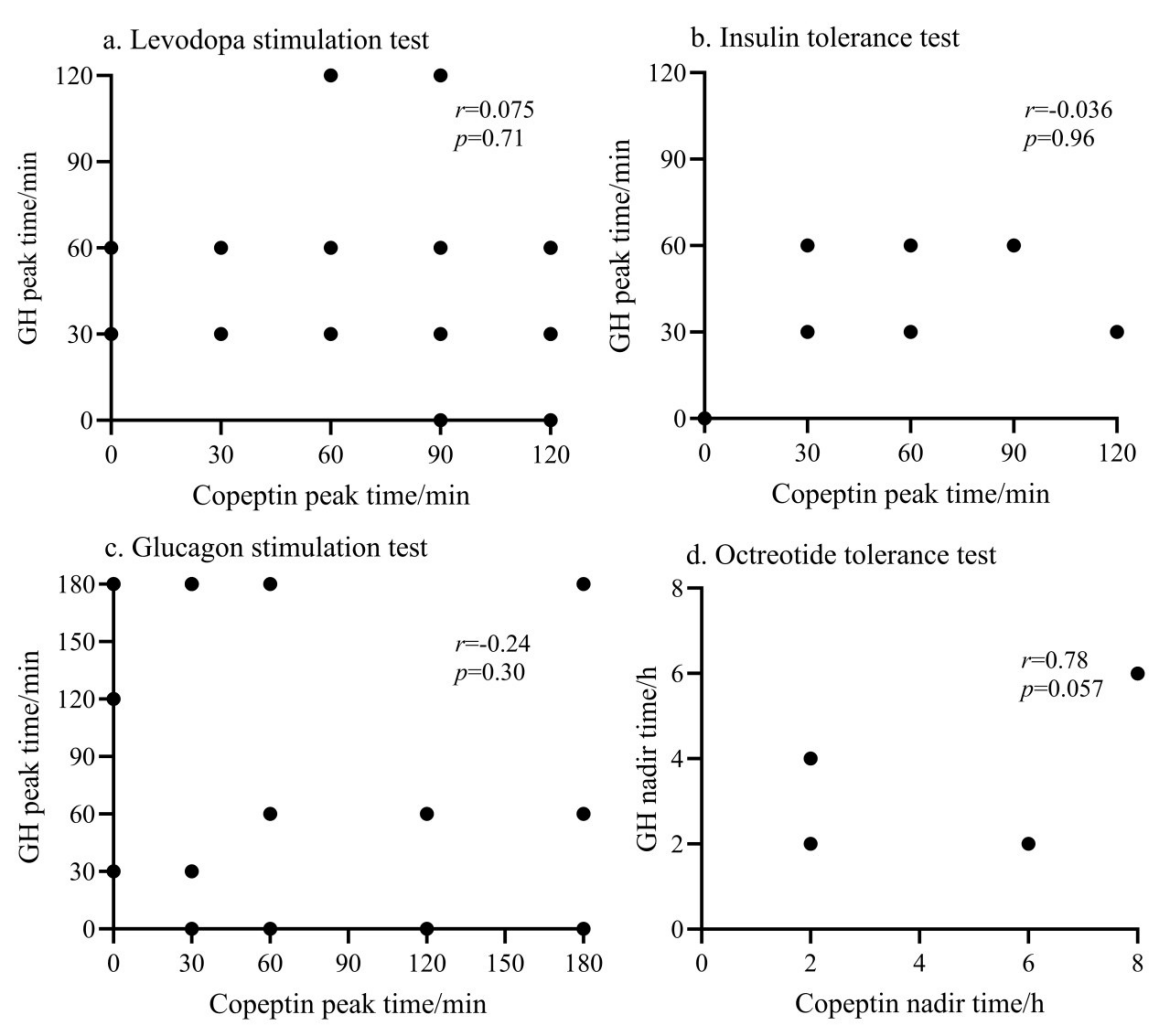
（a）左旋多巴激发试验（*n*=28）、（b）胰岛素低血糖兴奋试验（*n*=7）、（c）胰高血糖素激发试验（*n*=20）和（d）奥曲肽抑制试验（*n*=7）期间GH达峰时间与和肽素达峰时间之间的相关性

进一步探讨和肽素与GH水平变化之间的关系后，我们发现尽管4种刺激物均为GH功能试验的常规手段，但和肽素与GH在峰值、变化倍数以及达峰时间方面均无显著相关性。这表明两者虽然在垂体轴中可能存在一定关联，但其分泌调控机制并不完全一致，和肽素的变化更可能直接反映垂体后叶的功能状态，而GH主要由垂体前叶分泌。但是有研究显示，在儿童中，低血糖应激对和肽素的刺激反应可能与催乳素的激活有关，这提示应激诱导的和肽素释放与垂体前叶激活有关^［32］^。另一种假设是，AVP与CRH之间存在协同作用^［33］^，因此，AVP、和肽素和CRH可能在低血糖的应激状态下共同释放。

### 2.5 精氨酸加压素缺乏症患者在左旋多巴GH兴奋试验中和肽素水平变化

有4名进行了左旋多巴激发试验的患者被确诊为AVP-D，其临床数据见表5。AVP-D患者的和肽素最大值显著低于非AVP-D患者（*p*=0.000 2）。AUC为0.98（95% CI 0.94~1.00，*p*=0.002 1）。若采用31.10 pg/mL为诊断界值，则灵敏度为100%（95% CI 51.01%~100%），特异度为96.43%（95% CI 82.29%~99.82%）。ROC曲线结果显示，使用LC-MS/MS法检测和肽素也能实现AVP-D患者和非AVP-D患者的鉴别诊断。

**表 5:** 精氨酸加压素缺乏症患者的临床特征

No.	Sex	Age	*C*_（Copeptin）_/（pg/mL）					*C*_（GH）_/（ng/mL）				
			0 min	30 min	60 min	90 min	120 min	0 min	30 min	60 min	90 min	120 min
1	F	61	0	1.91	1.55	3.67	3.43	0.1	0.1	0.1	0.1	0.3
2	M	42	7.07	30.5	17.9	13	14	0.3	0.2	0.2	0.3	0.4
3	F	10	8.91	12.7	9.65	12.8	17.1	0.1	0.1	0.2	0.3	0.1
4	M	13	2.69	8.19	7.08	10.6	8.16	0.1	1.8	1.3	0.4	0.9

F： female； M： male.

Refardt等^［21］^的研究显示，高渗盐水刺激优于精氨酸输注试验，以3.8 pmol/L为诊断界值时，精氨酸输注试验鉴别AVP-D和PP的诊断准确率为74.4%；以4.9 pmol/L为诊断界值时，高渗盐水输注试验的诊断准确率为95.6%。Gippert等^［26］^的研究显示，胰岛素低血糖激发试验中，AUC为96.9%（95% CI 0.93~1.00），以3.0 pmol/L（12.06 pg/mL）为诊断界值时，灵敏度和特异度分别为91.7%和94.1%。值得注意的是，既往研究均使用免疫法检测和肽素^［3，7-9，21，26］^，而本研究所采用的LC-MS/MS方法在特异性以及抗干扰能力方面有较大优势。在多时间点采样的功能试验中，血样可能伴有溶血，影响免疫法检测的准确性。而LC-MS/MS通过质荷比识别目标肽段，更加特异；此外，前期干扰试验显示本文方法不受溶血、脂血和黄疸的干扰。本方法的检出限为2.5 pg/mL，灵敏度较高，线性范围广，具有良好的重复性，可为临床提供可靠的和肽素测定手段。未来应开展更大规模的前瞻性研究，以验证在左旋多巴和胰岛素刺激条件下，基于LC-MS/MS测定的和肽素在AVP-D患者中的诊断阈值。

## 3 结论

本研究基于实验室自建的LC-MS/MS方法，评估了左旋多巴、胰岛素、胰高血糖素和奥曲肽对和肽素分泌的影响。左旋多巴、胰岛素和胰高血糖素能够刺激和肽素的分泌，其中左旋多巴的效果最明显；而奥曲肽则会抑制其分泌。LC-MS/MS法通过其高灵敏度、高特异性和抗溶血干扰等优点，为PPS的诊断提供了方法学上的替代手段。

本研究的意义不仅在于观察到左旋多巴、胰岛素等刺激物对和肽素分泌的显著影响，更在于为AVP合成分泌功能评估探索了潜在的新型刺激方案。目前临床用于鉴别AVP-D与PP的功能试验多依赖高渗盐水或精氨酸等刺激，但这些方案存在需静脉给药、操作复杂、耐受性差等局限。在本研究中，口服左旋多巴作为一种安全、简便、耐受性良好的口服刺激手段，在大多数受试者中引起了显著的和肽素升高，并成功区分了AVP-D与非AVP-D患者，提示较为良好的诊断性能。

本研究存在一定局限性。首先，纳入的受试者样本量相对较小，其中胰岛素低血糖试验与奥曲肽敏感试验仅纳入7名受试者，样本量相对有限，这在一定程度上影响了统计分析的效能和结论的稳健性。基于此，我们计划未来开展更大规模的前瞻性研究，进一步验证本研究中观察到的结果，并尝试建立刺激后和肽素参考范围或诊断阈值，从而推动该方法在临床中的推广应用。其次，尽管LC-MS/MS在分析性能上有优势，但检测流程复杂，耗时较长，成本较高，不利于常规临床推广。未来可尝试优化前处理流程，或开发基于质谱原理的自动化平台，以提升检测效率、降低成本。此外，奥曲肽对和肽素分泌的抑制机制尚不明确，可能涉及不同的神经体液调节通路，值得进一步开展动物模型或机制性研究予以验证。综上，未来有必要在更大样本量的前瞻性研究中，进一步验证这些刺激物在和肽素激发试验中的敏感性、特异性及临床适用性，从而为PPS等疾病诊断方案提供新的技术选择。
